# Effectiveness of online tobacco control education: A cross-sectional study among healthcare professionals

**DOI:** 10.18332/tid/209148

**Published:** 2025-10-07

**Authors:** Yu Chen, Si Chen, Jing Xu, Li Xu, Ziliang Wang, Shiyu Liu, Yujiang Cai, Zining Wang, Xinjie Zhao, Xinyao Yu, Xinrui Yang, Na Zhang, Kin-Sun Chan

**Affiliations:** 1 School of Art and Communication, Fujian Polytechnic Normal University, Fuzhou, China; 2 China National Center for Food Safety Risk Assessment, Beijing, China; 3 School of Journalism and Communication, Peking University, Beijing, China; 4 Changchun Health Education Center, Changchun, China; 5 School of Public Health, Peking University, Beijing, China; 6 School of Public Health, Xi’an Jiaotong University, Xi'an, China; 7 School of International Studies, Peking University, Beijing, China; 8 Faculty of Humanities and Arts, Macau University of Science and Technology, Macao SAR, China; 9 Faculty of Social Sciences, University of Macau, Macao SAR, China; 10 School of Nursing, Hong Kong Polytechnic University, Hong Kong SAR, China

**Keywords:** MOOC, tobacco control, digital health education, healthcare workers

## Abstract

**INTRODUCTION:**

Online education platforms offer promising solutions for tobacco control capacity building. This study evaluated an online tobacco control course's effectiveness on healthcare professionals' knowledge, attitudes, and behavioral intentions.

**METHODS:**

A cross-sectional survey was conducted among healthcare workers and medical students (n=719) in a Chinese city, January 2023. Participants were categorized as course participants (n=387) or non-participants (n=332). The validated survey instrument (Cronbach's α=0.963) assessed tobacco-related knowledge, attitudes, and behavioral intentions using 5-point Likert scales. Statistical analyses included t-tests, effect size, and multivariable regression.

**RESULTS:**

Course participants demonstrated significantly higher knowledge scores across multiple domains compared to non-participants. Regarding specific tobacco harms, participants showed greater awareness that smoking causes stroke (4.21 ± 0.90 vs 3.86 ± 1.04, p<0.001, Cohen's d=0.37), heart disease (4.27 ± 0.85 vs 3.93 ± 1.03, p<0.001, d=0.36), and erectile dysfunction (4.05 ± 0.97 vs 3.72 ± 1.12, p<0.001, d=0.32). For secondhand smoke, participants better recognized risks of adult cardiovascular disease (4.26 ± 0.81 vs 4.04 ± 0.90, p=0.001, d=0.26) and pediatric respiratory illness (4.37 ± 0.73 vs 4.15 ± 0.83, p<0.001, d=0.28). Participants also showed more positive attitudes toward tobacco control policies and greater behavioral intentions for tobacco control advocacy. In multivariable analysis adjusting for demographics and smoking status, course participation remained significantly associated with higher knowledge scores (β=0.28; 95% CI: 0.18–0.38, p<0.001), more positive attitudes (β=0.22; 95% CI: 0.12–0.32, p<0.001), and stronger behavioral intentions (β=0.31; 95% CI: 0.19–0.43, p<0.001).

**CONCLUSIONS:**

The online tobacco control course significantly improved participants' knowledge of tobacco harms and strengthened their support for tobacco control measures. These findings suggest that digital health education platforms may be valuable tools for tobacco control capacity building, though further longitudinal studies are needed to establish causal relationships and assess long-term effectiveness.

## INTRODUCTION

Tobacco use remains one of the leading preventable causes of death globally, with China bearing a substantial burden as the world’s largest tobacco consumer and producer^1^. The most recent 2024 China National Adult Tobacco Survey (NATS) revealed that current smoking prevalence among Chinese adults aged ≥15 years was 23.2%, with pronounced gender disparities^2^. In 2021, tobacco was responsible for an estimated 2.7 million deaths in China, accounting for 34.6% of total deaths^3^. Healthcare professionals play a pivotal role in tobacco control implementation, yet systematic training programs remain limited and geographically uneven across China^4^.

Traditional tobacco control education faces multiple challenges, including resource constraints, geographical barriers, and limited scalability^5^. The emergence of Massive Open Online Courses (MOOCs) offers promising solutions for addressing these limitations, providing accessible, standardized, and cost-effective educational platforms^6^. MOOCs have demonstrated effectiveness in various health education domains, with advantages including flexible scheduling, repeatability, and broad reach^7^.

The Knowledge-Attitude-Practice (KAP) model provides a theoretical framework for understanding how educational interventions influence health behaviors^8^. This model posits that knowledge acquisition leads to attitude change, which subsequently influences behavioral intentions and practices^9^. Self-efficacy theory further emphasizes the importance of confidence in one’s ability to perform specific behaviors, serving as a crucial mediator between knowledge and action^10^.

Despite growing interest in digital health education, rigorous evaluations of online tobacco control training programs remain scarce, particularly in developing countries^11^. Recent studies indicate that healthcare workers in China continue to have substantial tobacco control education needs, with nearly one-third of male physicians estimated to be smokers as of 2023^12^. China’s particular tobacco control challenges, including high smoking prevalence among healthcare workers and cultural acceptance of tobacco use, necessitate culturally adapted educational approaches^12^.

Current evidence suggests that tobacco-related health education is associated with better smoking harm awareness and reduced secondhand smoke exposure among employees^13^. However, the effectiveness of online tobacco control education platforms specifically targeting healthcare professionals requires systematic evaluation. A 2021 cross-sectional study among 1028 respiratory healthcare workers from 89 hospitals in Fujian Province found that only 40.0% of participants were aware of the Healthy China 2030 tobacco control targets, highlighting significant knowledge gaps and the need for comprehensive training programs^14^.

This study aims to evaluate the effectiveness of an online tobacco control course using the KAP framework enhanced with self-efficacy measures. Specifically, we examine differences in tobacco-related knowledge, attitudes, and behavioral intentions between course participants and non-participants among healthcare workers and medical students in China.

## METHODS

### Study design and setting

This cross-sectional survey was conducted in January 2023 among healthcare workers and medical students in a middle-sized Chinese city with diverse healthcare institutions, providing a suitable setting for evaluating tobacco control educational interventions.

### Participants

Eligible participants included healthcare professionals and medical students working or studying within the city’s health system. Inclusion criteria were: 1) age ≥18 years; and 2) current employment or enrollment in healthcare/medical education institutions. Exclusion criteria were incomplete survey responses or evidence of systematic response patterns.

Participants were recruited through convenience sampling via the city’s health system communication channels. Healthcare professionals were identified through hospital staff directories, while medical students were recruited through medical school networks. Evidence of systematic response patterns was defined as identical responses across all scale items or completion time <30 seconds, indicating insufficient engagement with survey content.

### Sample size calculation

Sample size calculation was based on expected medium effect size (Cohen’s d=0.4) for knowledge differences between groups, with α=0.05 and power=0.80. This yielded a minimum requirement of 200 participants per group. Accounting for 20% incomplete responses, we targeted 500 total participants.

### Survey instrument

The survey instrument was adapted from established questionnaires including the Global Adult Tobacco Survey and China National Adult Tobacco Survey (NATS)^15,16^. The questionnaire underwent content validation by tobacco control experts and pilot testing among 50 healthcare workers. This study employed the KAP theoretical framework to examine how online education influences knowledge, attitudes, and behavioral intentions.

The final instrument comprised four domains: demographics and tobacco use status (10 items), tobacco-related knowledge covering smoking harms, secondhand smoke effects, and nicotine addiction (11 items), attitudes and beliefs assessing tobacco control support and personal relevance (8 items), and behavioral intentions and self-efficacy varying by smoking status and covering cessation intentions, advocacy behaviors, and confidence measures.

All knowledge, attitude, and behavior items used 5-point Likert scales (1 = ‘strongly disagree’ to 5 = ‘strongly agree’). The questionnaire demonstrated excellent internal consistency (Cronbach’s α=0.963) and adequate split-half reliability (Guttman coefficient=0.844).

### Data collection

Data were collected via online self-administered questionnaires distributed through the city’s health system communication channels. Participants provided informed consent before beginning the survey. Quality control measures included minimum completion time requirements (>30 seconds) and detection of systematic response patterns.

### Statistical analysis

Data were analyzed using SPSS 28.0. Descriptive statistics included frequencies and percentages for categorical variables and means with standard deviations for continuous variables. Missing data from invalid submissions were excluded from the analysis. The primary exposure variable was course participation (participants vs non-participants). Main outcomes were tobacco-related knowledge, attitudes, and behavioral intentions measured on 5-point Likert scales.

Group comparisons employed independent samples t-tests for continuous variables and chi-squared tests for categorical variables. Before conducting t-tests, normality was assessed using Shapiro-Wilk tests and Q-Q plots for samples <50 and visual inspection of histograms for larger samples. Homogeneity of variance was evaluated using Levene’s test. For variables violating normality assumptions, Mann-Whitney U tests were used as alternatives. Effect sizes were calculated using Cohen’s d, with values of 0.2, 0.5, and 0.8 representing small, medium, and large effects, respectively^17^. Cohen’s d with 95% confidence intervals were calculated to quantify effect sizes.

Multivariable linear regression models were constructed based on theoretical considerations from the KAP framework and statistical significance in univariate analyses. Variables with p<0.20 in univariate analysis and those considered theoretically important (age, gender, education level, smoking status) were included in the initial model, followed by backward elimination based on statistical significance and theoretical relevance. The association between course participation and outcomes was examined while controlling for potential confounders including age, gender, education level, income, and smoking status. Model assumptions were verified through residual analysis. Statistical significance was set at p<0.05 (two-tailed).

### Ethics statement

This study was approved by Peking University Biomedical Ethics Committee (IRB00001052-20056). All participants provided informed consent, and data were anonymized to protect privacy.

## RESULTS

### Participant characteristics

A total of 750 questionnaires were distributed, with 719 complete responses after excluding 31 invalid submissions, yielding a response rate of 95.9%. The analytical sample comprised 387 course participants (53.8%) and 332 non-participants (46.2%).

Participant demographics are presented in Table 1. The sample included 259 males (36.0%) and 460 females (64.0%), with mean age of 36.2 ± 11.4 years. Most participants (58.6%) worked in public health or medical systems, and 47.0% had college/undergraduate education. Current smoking prevalence was 17.8% (n=128), with significant differences between groups (13.95% vs 22.29%, p<0.001).

**Table 1:** Participant characteristics by course participation status (N=719)

*Characteristics*	*Course* *participants* *(N=387)* *n (%)*	*Non-participants* *(N=332)* *n (%)*	p*
**Age** (years)			0.023
18–25	49 (12.7)	129 (38.9)	
26–35	89 (23.0)	87 (26.2)	
36–45	100 (25.8)	74 (22.3)	
46–55	133 (34.4)	36 (10.8)	
≥56	15 (3.9)	5 (1.5)	
**Gender**			0.003
Male	122 (31.5)	137 (41.3)	
Female	265 (68.5)	195 (58.7)	
**Education level**			<0.001
High school or lower	91 (23.5)	91 (27.4)	
College/undergraduate	170 (43.9)	168 (50.6)	
Graduate degree	126 (32.6)	73 (22.0)	
**Smoking status**			0.003
Current smoker	54 (13.95)	74 (22.29)	
Former smoker	17 (4.39)	9 (2.71)	
Never smoker	316 (81.65)	249 (75.00)	

* p-values from chi-squared tests (categorical variables) and t-tests (continuous variables).

### Tobacco-related knowledge

Course participants demonstrated significantly higher knowledge across multiple tobacco harm domains (Table 2). For smoking-related harms, participants showed greater awareness that smoking causes stroke (4.21 ± 0.90 vs 3.86 ± 1.04, p<0.001, d=0.37), heart disease (4.27 ± 0.85 vs 3.93 ± 1.03, p<0.001, d=0.36), lung cancer (4.42 ± 0.80 vs 4.22 ± 0.85, p=0.002, d=0.24), erectile dysfunction (4.05 ± 0.97 vs 3.72 ± 1.12, p<0.001, d=0.32), and periodontitis (4.10 ± 0.92 vs 3.95 ± 0.92, p=0.030, d=0.16).

**Table 2:** Tobacco-related knowledge by course participation status

*Knowledge item*	*Course participants* *(N=387)* *Mean ± SD*	*Non-participants* *(N=332)* *Mean ± SD*	*Cohen’s* *effect size d*	p*
**Smoking harms**				
Stroke	4.21 ± 0.90	3.86 ± 1.04	0.37	<0.001
Heart disease	4.27 ± 0.85	3.93 ± 1.03	0.36	<0.001
Lung cancer	4.42 ± 0.80	4.22 ± 0.85	0.24	0.002
Erectile dysfunction	4.05 ± 0.97	3.72 ± 1.12	0.32	<0.001
Periodontitis	4.10 ± 0.92	3.95 ± 0.92	0.16	0.030
**Secondhand smoke harms**				
Adult cardiovascular disease	4.26 ± 0.81	4.04 ± 0.90	0.26	0.001
Pediatric respiratory illness	4.37 ± 0.73	4.15 ± 0.83	0.28	<0.001
Adult lung cancer	4.39 ± 0.74	4.17 ± 0.84	0.28	<0.001
Sudden infant death syndrome	4.23 ± 0.85	4.01 ± 0.96	0.24	0.001
**Addiction knowledge**				
Nicotine highly addictive	4.32 ± 0.82	4.19 ± 0.84	0.16	0.044

* p-values from t-tests. Likert scale: 1 = ‘strongly disagree’ to 5 = ‘strongly agree’.

Regarding secondhand smoke harms, participants better recognized risks including adult cardiovascular disease (4.26 ± 0.81 vs 4.04 ± 0.90, p=0.001, d=0.26), pediatric respiratory illness (4.37 ± 0.73 vs 4.15 ± 0.83, p<0.001, d=0.28), adult lung cancer (4.39 ± 0.74 vs 4.17 ± 0.84, p<0.001, d=0.28), and sudden infant death syndrome (4.23 ± 0.85 vs 4.01 ± 0.96, p=0.001, d=0.24).

**Figure 1:**
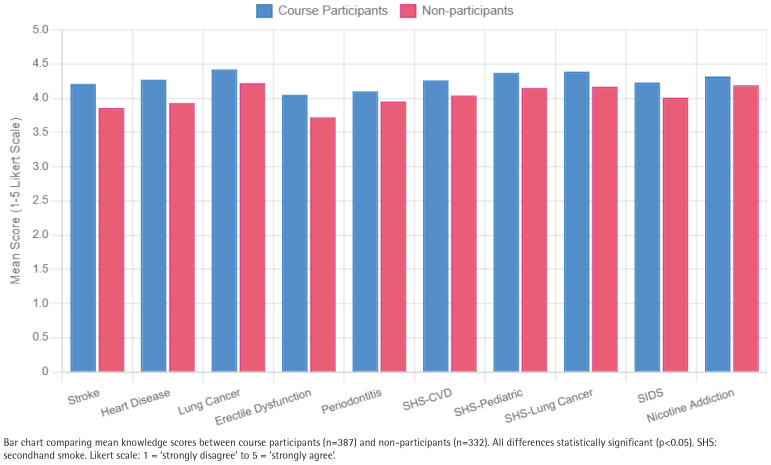
Tobacco-related knowledge scores by course participation status

Participants also showed greater understanding of nicotine addiction, with higher agreement that nicotine is highly addictive and difficult to quit (4.32 ± 0.82 vs 4.19 ± 0.84, p=0.044, d=0.16). These knowledge differences are visualized in Figure 1, showing consistent advantages for course participants across all domains.

### Attitudes and beliefs

Course participants demonstrated more positive attitudes toward tobacco control across all measured domains (Table 3). They showed greater agreement that tobacco control was relevant to their personal lives (4.40 ± 0.80 vs 4.16 ± 0.85, p<0.001, d=0.29) and work/study (4.34 ± 0.89 vs 4.07 ± 0.91, p<0.001, d=0.30). Participants expressed greater concern about smoking harms to personal health (4.45 ± 0.72 vs 4.27 ± 0.79, p=0.002, d=0.24).

**Table 3:** Tobacco control attitudes and beliefs by course participation status

*Attitude item*	*Course participants* *(N=387)* *Mean ± SD*	*Non-participants* *(N=332)* *Mean ± SD*	*Cohen’s* *effect size d*	p*
**Beliefs**				
Tobacco control relevant to life	4.40 ± 0.80	4.16 ± 0.85	0.29	<0.001
Tobacco control relevant to work/study	4.34 ± 0.89	4.07 ± 0.91	0.30	<0.001
Concern about smoking health harms	4.45 ± 0.72	4.27 ± 0.79	0.24	0.002
**Policy attitudes**				
Support comprehensive smoke-free policies	4.48 ± 0.72	4.30 ± 0.85	0.23	0.002
Support other tobacco control policies	4.47 ± 0.70	4.30 ± 0.79	0.23	0.002
Support national tobacco control initiatives	4.48 ± 0.70	4.33 ± 0.78	0.20	0.005

* p-values from t-tests. Likert scale: 1 = ‘strongly disagree’ to 5 = ‘strongly agree’.

Support for tobacco control policies was consistently higher among course participants, including support for comprehensive smoke-free policies in public places, workplaces, and transportation (4.48 ± 0.72 vs 4.30 ± 0.85, p=0.002, d=0.23), other tobacco control policies (4.47 ± 0.70 vs 4.30 ± 0.79, p=0.002, d=0.23), and national tobacco control initiatives (4.48 ± 0.70 vs 4.33 ± 0.78, p=0.005, d=0.20).

**Figure 2:**
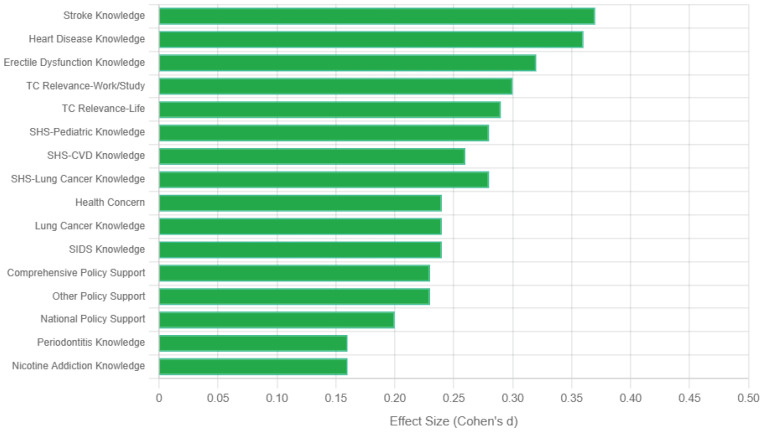
Cohen’s effect size for knowledge, attitudes and behavioral intentions

Effect sizes for all attitude and policy support measures are presented in Figure 2, demonstrating consistent small to medium effects favoring course participants.

### Behavioral intentions and self-efficacy

Course participants reported higher intentions for tobacco control advocacy behaviors across multiple domains. They were more likely to report intentions to complain about indoor smoking (2.88 ± 2.83 vs 1.88 ± 3.25, p<0.001), advise others to quit smoking (3.04 ± 2.88 vs 1.95 ± 3.29, p<0.001), discourage smoking in their homes (3.06 ± 2.90 vs 2.00 ± 3.32, p<0.001), and discuss tobacco harms with family members (smoking: 3.16 ± 2.93 vs 2.08 ± 3.36, p<0.001; secondhand smoke: 3.18 ± 2.93 vs 2.12 ± 3.38, p<0.001).

Professional tobacco control engagement was also higher among participants, including discussing tobacco control with colleagues (3.12 ± 2.90 vs 2.03 ± 3.33, p<0.001), focusing on tobacco control at work (3.13 ± 2.91 vs 2.05 ± 3.34, p<0.001), and actively promoting tobacco control efforts (3.14 ± 2.91 vs 2.04 ± 3.34, p<0.001).

Self-efficacy measures varied by smoking status. Among non-smokers, course participants reported greater confidence in remaining smoke-free (4.73 ± 1.41 vs 4.47 ± 1.93, p=0.036) and refusing offered cigarettes (4.73 ± 1.41 vs 4.46 ± 1.93, p=0.035). Among current smokers, participants showed greater intentions to consider quitting (2.55 ± 1.75 vs 2.25 ± 2.19, p=0.043) and attempt cessation (2.55 ± 1.75 vs 2.24 ± 2.21, p=0.040).

### Multivariable analysis

After adjusting for demographics, smoking status, and other potential confounders, course participation remained significantly associated with higher knowledge scores (β=0.28; 95% CI: 0.18–0.38, p<0.001), more positive attitudes (β=0.22; 95% CI: 0.12–0.32, p<0.001), and stronger behavioral intentions (β=0.31; 95% CI: 0.19–0.43, p<0.001) (Table 4).

**Table 4:** Multivariable regression analysis*

*Outcome*	*Course* *participation* *β (95% CI)*	*p*	*Adjusted* *R^2^*
Knowledge score	0.28 (0.18–0.38)	<0.001	0.156
Attitude score	0.22 (0.12–0.32)	<0.001	0.132
Behavioral intentions	0.31 (0.19–0.43)	<0.001	0.198

* Models adjusted for age, gender, education level, income, and smoking status. β: standardized coefficient.

## DISCUSSION

This study evaluated an online tobacco control course, demonstrating significant positive effects on participants’ tobacco-related knowledge, attitudes, and behavioral intentions. The findings support the effectiveness of digital health education platforms for tobacco control capacity building among healthcare professionals.

### Principal findings

Course participants showed substantially improved knowledge across multiple tobacco harm domains, with effect sizes ranging from small to medium. These improvements were particularly pronounced for specific smoking-related harms including cardiovascular and reproductive health effects, areas that have received less attention in traditional anti-tobacco messaging focused primarily on lung cancer^18^. Enhanced awareness of secondhand smoke harms, especially risks to children, may be particularly valuable given China’s high rates of household smoke exposure^19^.

The observed improvements in attitudes and policy support are noteworthy given the challenging tobacco control environment in China, where tobacco industry influence and cultural acceptance of smoking remain substantial barriers^20^. Participants’ increased willingness to engage in tobacco control advocacy behaviors suggests potential for amplified impact as trained individuals become change agents within their professional and social networks.

### Mechanisms of effect

The observed improvements align with the KAP theoretical framework, suggesting that structured knowledge acquisition through the online course enhanced participants’ understanding of tobacco harms, which in turn influenced their attitudes and behavioral intentions^21^. The course’s comprehensive approach, covering both direct smoking harms and secondhand smoke effects, appears to have been particularly effective in raising awareness of tobacco’s broad health impacts.

Self-efficacy improvements, particularly among non-smokers for maintaining tobacco-free status and among smokers for cessation intentions, suggest that the course successfully enhanced participants’ confidence in tobacco control behaviors. This finding is consistent with social cognitive theory, which emphasizes self-efficacy as a crucial determinant of behavior change^22^.

### Implications for practice and policy

These findings have important implications for tobacco control efforts. The demonstrated effectiveness of online education suggests that digital platforms may serve as scalable solutions for national tobacco control capacity building. Given the vast healthcare workforce and geographical disparities in training access, digital platforms offer particular advantages for standardized, high-quality education delivery^23^.

The course’s effectiveness among healthcare professionals is particularly valuable, as these individuals serve as credible sources of health information and role models for the general public^24^. Enhanced tobacco control knowledge and advocacy intentions among healthcare workers could contribute to broader social norm changes regarding tobacco use.

For policymakers, these results suggest that investment in digital health education infrastructure may complement comprehensive tobacco control strategies, though cost-effectiveness analyses and randomized controlled trials are needed to confirm optimal implementation approaches^25^.

### Strengths and limitations

This study’s strengths include its large sample size, validated instruments, and comprehensive assessment across the KAP framework. The comparison between course participants and non-participants provides evidence for effectiveness, while the multivariable analysis controls for potential confounders.

However, several limitations must be acknowledged. The cross-sectional design precludes causal inference and may be subject to reverse causality, whereby individuals with greater interest in tobacco control were more likely to participate in the course. Self-reported measures may introduce information bias and misclassification. Additionally, residual confounding from unmeasured variables such as motivation levels or prior tobacco control experience may have influenced the results. The study’s geographical limitation may limit generalizability to other Chinese regions with different tobacco control contexts.

Additionally, the convenience sampling approach and the potential for volunteer bias among course participants may limit the representativeness of findings. Future research should employ randomized controlled designs to strengthen causal inference and include behavioral outcome measures to assess whether improved intentions translate into actual practice changes.

### Future research

Future studies should examine the persistence of knowledge and attitude improvements over time, as well as the translation of behavioral intentions into actual tobacco control practices. Cost-effectiveness analyses comparing online versus traditional training approaches would inform resource allocation decisions. Research on optimal course design elements and personalization strategies could further enhance educational effectiveness.

Investigation of online education’s impact on tobacco control climate within healthcare institutions would provide insights into broader organizational change processes. Additionally, studies examining the effectiveness of similar online education approaches for other health topics could inform digital health education strategy more broadly.

## CONCLUSIONS

This evaluation demonstrates that an online tobacco control course significantly improved participants’ tobacco-related knowledge, attitudes, and behavioral intentions. The findings suggest that digital health education platforms may be valuable tools for tobacco control capacity building, though further longitudinal studies are needed to establish causal relationships and assess long-term effectiveness. As countries continue to strengthen tobacco control implementation, online education platforms may offer useful tools for scaling training to healthcare professionals, though additional research is needed to confirm their effectiveness and optimal implementation strategies.

The success of this approach provides a model for other developing countries facing similar tobacco control challenges and resource constraints. Digital health education platforms may serve as valuable complements to traditional training approaches, offering scalable solutions for building the professional capacity needed to advance global tobacco control goals.

## Data Availability

The data supporting this research are available from the authors on reasonable request.
